# The Basic Pathology and Specific Treatment of Diphtheria

**Published:** 1885-02

**Authors:** 


THE BASIC PATHOLOGY AND SPECIFIC TREATMENT OF DIPHTHERIA, TYPHOID, ZYMOTIC, SEPTIC, SCORBUTIC, AND PUTRESCENT DISEASES GENERALLY, by Geo. J. Ziegler, M. D.

If the author of this production had adopted the same mode of investigation, and not simply borne the same name with the great pathologist, a very different result ought to have been reached by his mental inferences from the data before him.

The facts connected with the elimination of ammonia by the various excretory processes and the separation of effete matters from the body, do not sustain the position that the presence of ammonia in the different organs and tissues is a source of disturbance to the organization; and there is sufficient evidence in favor of the salutary influence of the inhalation of ammonial vapors in the normal state and the internal use of this alkali in abnormal conditions, to set aside his reasoning. Notwithstanding the fact that excrementitious matters emit ammoniacal vapors largely and continuously—it is well known that the Dutch, who dwell over their cattle stalls and the Arabs who occupy the same quarters with their horses, are remarkably exempt from this class of diseases; and it is equally established that the volatile alkali is the most efficacious antidote to such poisoning and to other depressing and disintegrating diseases, while it is the remedy of all others, most efficacious in nervous rheumatism and in many derangements of the nervous system. It is more in accordance with a correct view of pathology, that the troubles which seem to have the common factor of loss of tone in the vital organization result from undue elimination of ammonia than from its preponderance in the system; and the prime element of this derangement is a disorder of the nerve centers, from depressing agencies.

The laudatory comments upon the efficacy of spirits of turpentine in adynamic and putrescent diseases, will be indorsed by all who have had any considerable experience with this agent. "It stimulates the heart and circulation, brain, nervous and general system, disinfects and depurates the blood and body, and acts as a general alterative, resolvent, stimulant and corroborant." This part of his treatment is sanctioned, but from a different stand-point.

